# Comparative Neurotoxic Effects of Doxorubicin and Sunitinib: An In Vitro Study on Human Dopaminergic Neuronal Cells

**DOI:** 10.3390/molecules30112342

**Published:** 2025-05-27

**Authors:** Maria Barbosa-Azevedo, Maria B. Igreja-Cardoso, Félix Carvalho, Vera Marisa Costa

**Affiliations:** 1 Associate Laboratory i4HB—Institute for Health and Bioeconomy, Faculty of Pharmacy, University of Porto, 4050-313 Porto, Portugalfelixdc@ff.up.pt (F.C.); 2 UCIBIO-Applied Molecular Biosciences Unit, Laboratory of Toxicology, Department of Biological Sciences, Faculty of Pharmacy, University of Porto, 4050-313 Porto, Portugal

**Keywords:** chemobrain, doxorubicin, sunitinib, autophagy, redox homeostasis

## Abstract

Chemotherapy-induced cognitive impairment, commonly referred to as chemobrain, is a well-documented adverse outcome of anticancer treatments. While the neurotoxicity of doxorubicin (DOX) has been extensively studied, targeted therapies such as sunitinib (SUN) remain largely unexplored concerning this outcome. This study aimed to compare the neurotoxic effects of DOX and SUN in dopaminergic neuronal cells and to explore the involvement of oxidative stress and autophagy as potential mechanisms underlying their cytotoxicity. Human neuronal SH-SY5Y cells were differentiated into a dopaminergic phenotype and exposed to clinically relevant concentrations of DOX (0.1–10 µM) and SUN (1–10 µM) for 24 or 48 h. To investigate the involvement of oxidative stress in their cytotoxicity, redox modulators [N-acetylcysteine (NAC); dimethyl fumarate (DMF); sulforaphane (SFN); and cheirolin (CH)] were tested alongside DOX and SUN for their potential protective effects. The role of autophagy in SUN-induced toxicity was assessed using 3-methyladenine (3-MA; an early-stage inhibitor); chloroquine (CH; a late-stage inhibitor); and rapamycin (RAP; an autophagy inducer). Additionally, LC3-I and LC3-II expression levels were determined. Both DOX and SUN exhibited time- and concentration-dependent cytotoxicity and induced mitochondrial membrane depolarization. NAC conferred partial protection against SUN toxicity but enhanced DOX’s cytotoxicity at the lowest concentration tested. DMF and SFN had dual effects, depending on the drug’s concentration, while CH exhibited a consistent protective effect towards the cytotoxicity induced by both drugs. Regarding autophagy, 3-MA partially protected against SUN-induced toxicity, whereas CLQ and RAP exacerbated it. LC3-II levels were increased in some conditions, suggesting that SUN-induced toxicity involves autophagy. This study shows that SUN, though less studied in chemobrain, has a cytotoxic profile similar to DOX, which is a known contributor to chemobrain, in SH-SY5Y cells. These findings highlight the need for further research on neuroprotective strategies targeting oxidative stress and autophagy to reduce chemobrain in cancer patients and survivors.

## 1. Introduction

According to the Global Cancer Observatory, approximately 20 million new cancer cases and 9.7 million cancer-related deaths occurred worldwide in 2022 [1]. Despite this burden, the 5-year survival rate has improved significantly since the mid-1970s due to advances in cancer prevention, early detection, and treatment [2,3]. However, increased survival rates have also brought attention to the long-term adverse effects of cancer treatments, particularly their impact on cognitive function. The term “chemobrain” (or “chemotherapy-induced cognitive impairments”) describes cognitive dysfunction resulting from the neurotoxic effects of anticancer treatments on the central nervous system (CNS) [4].

Doxorubicin (DOX), also known as adriamycin, is one of the most effective chemotherapeutic agents. It is used to treat a range of cancers, including solid tumors, such as breast and liver cancer, as well as hematological malignancies [5,6]. A meta-analysis revealed a cognitive decline in DOX-treated breast cancer patients, when compared to healthy controls, with short-term verbal memory being the most affected cognitive domain, alongside impairments in executive function and processing speed [7]. In vivo animal studies also report cognitive impairment following DOX administration [8,9,10,11]. DOX is commonly believed to be unable to cross the blood–brain barrier (BBB). However, a clinical study by Stewart and colleagues detected DOX (0–58 ng/g) and its metabolite, doxorubicinol (DOXOL) (0–191 ng/g), in the brain of DOX-treated patients [12]. Animal studies have also reported low but detectable DOX levels in the cerebellum following intraperitoneal administration (0.26 ± 0.09 ng/mg fresh tissue) [13]. Regardless of the ongoing debate on whether DOX can cross the BBB, oxidative stress is considered a key mechanism underlying DOX-induced chemobrain. DOX has been shown to trigger lipid peroxidation, elevate nitric oxide and mitochondrial H_2_O_2_ levels in the hippocampus, and deplete essential antioxidants such as glutathione (GSH) and catalase [14,15,16,17]. Consequently, oxidative stress in the brain can lead to mitochondrial dysfunction, glial cell activation, programmed cell death, and proteasomal impairment [18]. Together with oxidative stress, inflammation also plays a role in DOX-induced neurotoxicity. Aluise et al. found that DOX oxidizes ApoA-I in plasma, impairing its ability to suppress TNF-α release [19], leading to increased serum TNF-α levels. Inflammation can disrupt BBB integrity, allowing pro-inflammatory molecules to enter the brain and exacerbate neuroinflammation, further triggering oxidative stress [20,21].

Sunitinib (SUN) is a targeted therapy initially developed to inhibit cancer cell growth by selectively targeting and disrupting the activity of specific molecules essential for tumor development and carcinogenesis [22,23]. SUN is a tyrosine kinase inhibitor (TKI) and it was approved in first-line treatment for patients with advanced renal cell carcinoma and for the treatment of patients with gastrointestinal stromal tumors after disease progression or intolerance to imatinib mesylate therapy [24]. SUN inhibits several kinases, including vascular endothelial growth factor receptors (VEGFR 1, 2, 3) and platelet-derived growth factor receptors (PDGFR α, β) [25,26,27]. Like DOX, SUN also exhibits poor brain penetration [28]. However, some studies suggest that SUN can cross the BBB due to its physicochemical properties and through organic anion transporters 1B1 and B3 (ATP1B1 and OATP1B3) [29,30]. Compared to DOX, SUN has been less extensively studied concerning chemobrain. However, van der Veld et al. reported cognitive impairment induced by SUN administration [31]. In this study, three patients developed cognitive disorders upon SUN treatment, all of whom recovered within a week, which is approximately the elimination half-life of SUN. This supports the link between cognitive impairments and SUN treatment [31]. Mulder and colleagues, who found that patients receiving SUN treatment presented a poorer performance in cognitive tests, had similar results [32]. Moreover, patients undergoing longer SUN treatment showed poorer working memory performance compared to those with shorter treatment durations, though long-term clinical follow-up was not conducted [32]. Other TKIs, like dasatinib have been linked to verbal memory issues and cognitive complaints, though objective testing shows only mild impairments. Imatinib and nilotinib are associated with fatigue and insomnia, which may indirectly affect cognition [33]. Focusing on SUN, a pre-clinical study has shown that SUN induces cognitive impairment in mice [34]. SUN treatment resulted in significant neurodegeneration in the hippocampus and cerebral cortex [34], which are areas critical for both learning and memory [35]. In the same study, SUN also led to alterations in chromatin condensation, mitochondrial damage, and accumulated autophagosomes in the neurons of SUN-treated mice [34]. As far as our literature search indicates, this is the only mechanistic study to explore the relationship between SUN and chemobrain. While oxidative stress is a well-established mechanism underlying DOX-induced neurotoxicity, evidence also suggests that SUN induces oxidative damage in non-neural tissues. A study in L02 cells (a human hepatocyte model) observed that SUN exposure triggered oxidative stress [36]. Bouitbir et al. reported increased H_2_O_2_ production in cardiac fibers from SUN-treated mice compared to controls [37]. However, no studies have specifically investigated the involvement of oxidative stress in SUN-induced chemobrain.

Therefore, this study aimed to compare the neurotoxicity of DOX and SUN using dopaminergic-differentiated SH-SY5Y cells as a model. The concentrations used for both drugs were based on clinically relevant levels found in the plasma and brain of treated patients [12,38,39]. To our knowledge, this is the first mechanistic study investigating SUN’s effects in human neuronal cells. Additionally, we assessed the role of redox homeostasis in the toxicity of these anticancer drugs using different redox homeostasis markers and evaluated the contribution of autophagy to SUN-induced toxicity.

## 2. Results

### 2.1. Doxorubicin and Sunitinib Caused Time- and Concentration- Dependent Cytotoxicity

**Figure 1:**
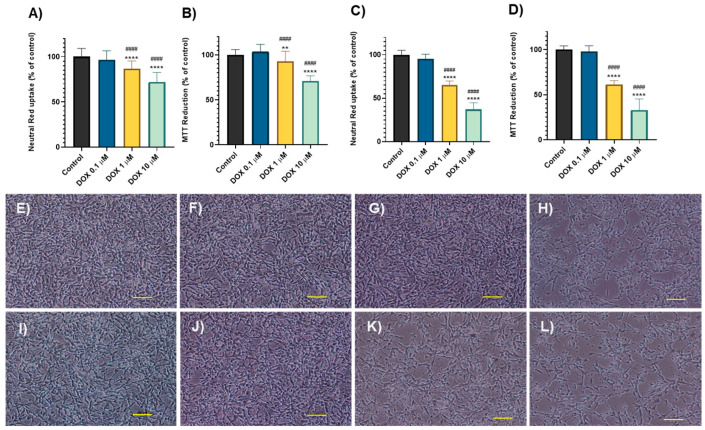
NR uptake and MTT reduction assays in differentiated SH-SY5Y cells exposed to 0.1, 1, or 10 μM DOX for 24 h (**A**,**B**) and 48 h (**C**,**D**). Data are presented as mean ± SD from 3–7 independent experiments (N = 18–42). Statistical analysis was conducted using a one-way ANOVA, followed by the Tukey’s post hoc test (** *p* < 0.01, **** *p* < 0.0001 vs. control; ^####^ *p* < 0.0001 vs. the concentration immediately below). Morphological analysis by phase contrast microscopy of differentiated SH-SY5Y cells after a 24/48-h exposure to DOX 0.1 μM (**F**,**J**), DOX 1 μM (**G**,**K**), or DOX 10 μM (**H**,**L**) using medium as the control group (**E**,**I**). Representative images of three independent experiments. Scale bare: 100 µm.

Cytotoxic effects were assessed using neutral red (NR) uptake and MTT reduction assays at clinically relevant concentrations (0.1–10 μM) of DOX at two time points: 24 h (Figure 1A,B) and 48 h (Figure 1C,D). The toxicity profile of DOX demonstrated a concentration- and time-dependent cytotoxicity in differentiated SH-SY5Y cells. At the lowest tested concentration (0.1 μM), DOX did not induce significant cytotoxicity at either 24 h (NR: 96.66 ± 9.86%, MTT: 103.5 ± 8.10%) or 48 h (NR: 95.21 ± 5.31%, MTT: 97.87 ± 6.33%) compared to the control groups. However, higher concentrations (1 and 10 μM) resulted in significant cytotoxic effects at both time points. After 24 h, DOX at 1 μM led to MTT reduction values of 86.75 ± 8.59% and NR uptake of 92.69 ± 5.67%. At 10 μM, cytotoxicity was more pronounced, with MTT reduction at 71.89 ± 10.81% and NR uptake at 71.00 ± 5.67%, compared to the control values (NR: 100 ± 9.01%, MTT: 100 ± 5.89%). Cytotoxicity was further intensified after 48 h, confirming a time-dependent increase in toxicity. At 1 μM, NR uptake and MTT reduction decreased to 65.12 ± 4.71% and 61.38 ± 4.12%, respectively. At the highest concentration (10 μM), MTT reduction dropped to 37.07 ± 7.73%, and NR uptake to 33.03 ± 12.34%, compared to the control groups (NR: 100 ± 5.12%, MTT: 100 ± 4.06%).

Regarding cells’ morphology, at 24 h, DOX at 0.1 µM (Figure 1F) and 1 µM (Figure 1G) did not induce significant cell damage, when compared to the control (Figure 1E). However, the highest concentration (DOX 10 µM) caused visible cell damage, with the cells acquiring a rounder shape and presenting fewer neurites (Figure 1H). With the increased exposure period (48 h), the cell damage was exacerbated. DOX at 1 µM (Figure 1K) induced significant cell damage, as indicated by the increased number of rounded cells compared to the control (Figure 1I). At the concentration of 10 µM (Figure 1L), the results showed comparable effects. Additionally, the control group exhibited a greater number of neurites, while cells treated with 1 µM and 10 µM of DOX had fewer. The lowest concentration tested (0.1 µM) did not induce any significant cell damage (Figure 1J) when compared to control cells.

**Figure 2:**
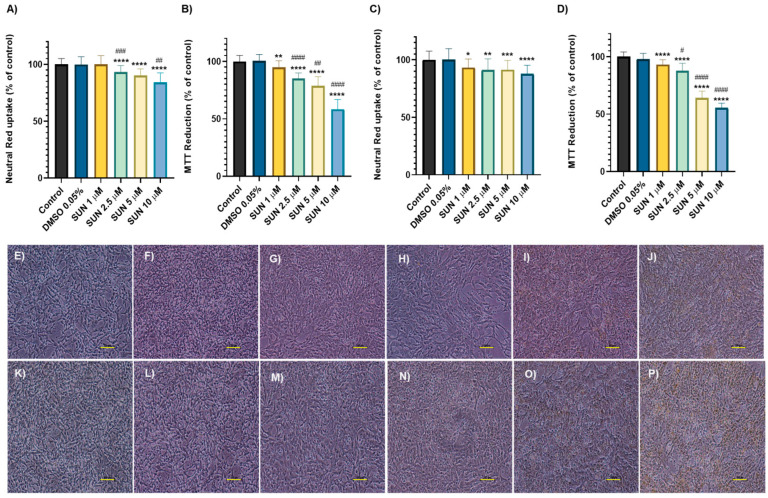
NR uptake and MTT reduction assays in differentiated SH-SY5Y cells exposed to 1, 2.5, 5, and 10 μM of SUN for 24 h (**A**,**B**) and 48 h (**C**,**D**). Data are presented as mean ± SD from 3–12 independent experiments (N = 18–72). Statistical analysis was conducted using a one-way ANOVA, followed by Tukey’s post hoc test (* *p* < 0.05, ** *p* < 0.01, *** *p* < 0.001, **** *p* < 0.0001 vs. control, ^#^ *p* < 0.05, ^##^ *p* < 0.01, ^###^ *p* < 0.001, ^####^ *p* < 0.0001 vs. the concentration immediately below). Morphological analysis by phase contrast microscopy of differentiated SH-SY5Y cells after a 24/48-h exposure to SUN 1 μM (**G**,**M**), SUN 2.5 μM (**H**,**N**), SUN 5 μM (**I**,**O**), or SUN 10 μM (**J**,**P**) using medium (**E**,**K**) as the control and DMSO 0.05% (**F**,**L**) as the vehicle. Representative images of three independent experiments. Scale bare: 100 µm.

Regarding SUN, this drug induced a time- and concentration-dependent cytotoxicity in differentiated SH-SY5Y cells. After 24 h, in the NR uptake assay the lowest incubation concentration (1 μM) did not show significant changes (100 ± 6.94%) compared to the control group (100 ± 5.14%) (Figure 2A). However, the higher concentrations tested presented significant cytotoxicity: 2.5 μM (93.32 ± 5.57%), 5 μM (90.30 ± 5.77%), and 10 μM (84.03 ± 8.54%). The MTT reduction assay revealed significant cytotoxicity across all concentrations of SUN (1 µM (94.98 ± 3.53%); 2.5 µM (85.20 ± 4.78%); 5 µM (78.63 ± 8.19%) and 10 µM (58.76 ± 8.36%)) (Figure 2B) compared to the control (100 ± 5.33%). These results reveal a clear dose-dependent toxicity. After 48 h, significant cytotoxicity was observed at all concentrations in both NR uptake and MTT reduction assays. For 1 μM, the results were NR: 93.25 ± 7.49% and MTT: 93.10 ± 4.30%, respectively; for 2.5 μM, 91.11 ± 9.76% and 87.82 ± 6.41%; for 5 μM, 91.36 ± 8.20% and 64.03 ± 5.94%; and for 10 μM, 87.75 ± 7.54% and 54.34 ± 5.22% (Figure 2C,D), compared to the respective controls. These results suggest a slight increase in toxicity over time, confirming a time-dependent cytotoxicity. The highest concentration of DMSO used as vehicle for SUN was 0.05% and it did not induce any significant changes at any of the tested time points or assays compared to the respective controls.

The morphology of the neuronal cells was also tested. At the 24-h time-point, all the tested concentrations of SUN caused significant cell damage to differentiated SH-SY5Y cells. For SUN, concentrations of 1 μM (Figure 2G) and 2.5 μM (Figure 2H) resulted in significant cell damage, as indicated by an increase in the number of rounded cells compared to the control (Figure 2E). At higher concentrations, 5 μM (Figure 2I) and 10 μM (Figure 2J), the extent of this damage was further amplified. Additionally, there was a progressive decrease in neurites with increasing concentrations of SUN. The cells exposed to SUN also presented yellow inclusions, which were more visible at the higher concentrations tested. The highest concentration of DMSO tested (0.05%) did not induce notable cellular damage (Figure 2F). The cellular damage was exacerbated with the longest exposure time, 48 h. SUN 1 μM (Figure 2M) and SUN 2.5 μM (Figure 2N) led to cellular damage, visible in the rounded shape of the cells, compared to the control (Figure 2K). Once again, this damage was even more visible in the highest concentrations of SUN (Figure 2O,P), where a decrease in the number of neurites was also seen. The yellow inclusions present in the cells exposed to SUN were evident, especially at the SUN 5 μM (Figure 2O) and SUN 10 μM (Figure 2P) conditions. The vehicle condition (DMSO 0.05%) did not induce any significant damage when compared to the controls (Figure 2L).

### 2.2. Doxorubicin- and Sunitinib-Induced Mitochondrial Membrane Depolarization

**Figure 3:**
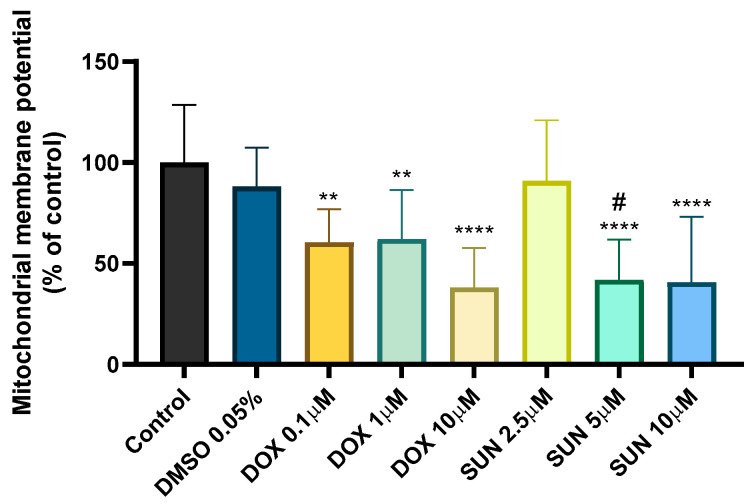
Mitochondrial membrane potential evaluation using the JC-1 probe in differentiated SH-SY5Y cells. Cells were incubated for 24 h with DOX (0.1 μM, 1 μM, and 10 μM) and SUN (2.5 μM, 5 μM, and 10 μM) for 24 h. The highest concentration of DMSO used for SUN (DMSO 0.05%) was also tested. The results are expressed as a percentage of the control cells and are mean ± SD of 2–5 independent experiments (7–21 wells). Statistical analysis was conducted using a one-way ANOVA, followed by Tukey’s post hoc test (** *p* < 0.01, **** *p* < 0.0001 vs. control; ^#^ *p* < 0.05 vs. SUN 2.5 μM).

Since SUN had a greater impact on the MTT reduction assay than the NR uptake assay, and DOX is a known mitochondrial toxin, Δψm was evaluated and compared in neuronal cells exposed to both drugs (Figure 3). A decrease in Δψm was observed at all tested concentrations of DOX (0.1 μM: 68.08 ± 30.55%; 1 μM: 62.06 ± 24.23%, and 10 μM: 43.03 ± 28.06%) and at SUN 5 μM (41.83 ± 19.94%) and SUN 10 μM (40.72 ± 32.48%) compared to the control (100 ± 28.57%) (Figure 3). The lowest concentration of SUN (2.5 μM) did not induce significant changes (90.84 ± 30.06%). The vehicle condition (DMSO 0.05%) was also tested, and no significant alterations were observed (88.12 ± 19.13%).

### 2.3. Dimethyl Fumarate, Sulphoraphane, and Cheirolin Conferred Dissimilar Outcomes Against the Toxicity Induced by Doxorubicin

**Figure 4:**
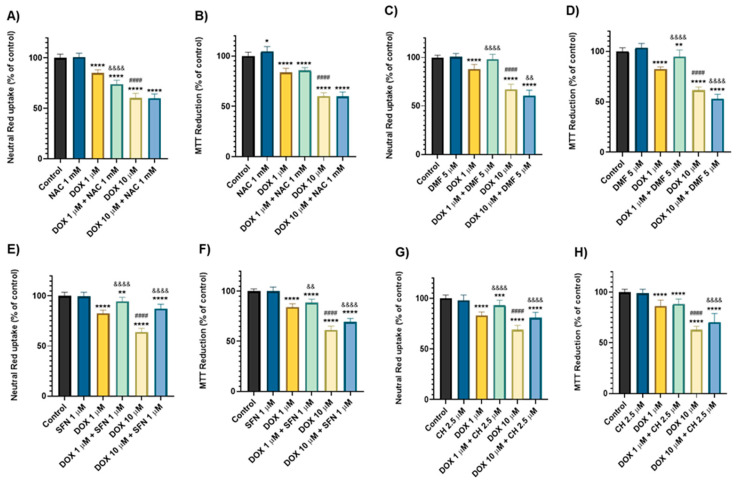
NR uptake and MTT reduction assays in differentiated SH-SY5Y cells exposed to 1 and 10 μM of DOX and NAC 1 mM (**A**,**B**), DMF 5 µM (**C**,**D**), SFN 1 µM (**E**,**F**) and CH 2.5 µM (**G**,**H**) for 24 h. Data are presented as mean ± SD from 3–4 independent experiments (N = 12–16). Statistical analysis was conducted using a one-way ANOVA, followed by the Tukey’s post hoc test (* *p* < 0.05, ** *p* < 0.01, *** *p* < 0.001, **** *p* < 0.0001 vs. control; ^####^ *p* < 0.0001 vs. the lower concentration of the anticancer drug; ^&&^ *p* < 0.01,^&&&&^ *p* < 0.0001 vs. the same concentration of the drug without the redox homeostasis modulator).

In the NR uptake assay, co-incubation with N-acetylcysteine (NAC) 1 mM increased the cytotoxicity of DOX (1 µM), leading to a decrease in NR uptake of 74.08 ± 3.84% compared to 85.15 ± 3.84% for DOX (1 µM) alone. At the highest concentration of DOX tested (10 µM), NAC did not produce significant changes (Figure 4A) when compared to the drug alone. In contrast, the MTT reduction assay showed no significant differences in toxicity between cells treated with DOX alone and those treated with DOX plus NAC (Figure 4B). Interestingly, this assay revealed that NAC alone increased MTT reduction levels in SH-SY5Y cells, with values of 104.6 ± 4.75%, compared to the control at 100 ± 3.77%.

Dimethyl fumarate (DMF) at 5 μM exhibited differential effects on DOX-induced cytotoxicity in differentiated SH-SY5Y cells, offering protection at lower DOX concentrations, while exacerbating toxicity at higher concentrations. In both the NR uptake and MTT reduction assays, DMF 5 μM mitigated the cytotoxicity of DOX 1 μM, resulting in values similar to the controls: NR: 98.06 ± 5.21% and MTT: 94.73 ± 6.77%, respectively, compared to DOX 1 μM alone (NR: 87.89 ± 4.97% and MTT: 81.78 ± 3.38%) (Figure 4C,D). Conversely, at a higher DOX concentration of 10 μM, DMF 5 μM increased its’ cytotoxicity. In the NR uptake assay, NR uptake decreased from 67.14 ± 5.29% to 60.60 ± 5.59% (Figure 4C). Similarly, in the MTT reduction assay, co-incubation with DMF 5 μM decreased the ability to reduce MTT from 61.57 ± 3.16% to 52.96 ± 4.60% (Figure 4D).

D,L-sulforaphane (SFN) at 1 µM protected differentiated SH-SY5Y cells against DOX-induced cytotoxicity. Both assays showed that SFN at 1 µM protected differentiated SH-SY5Y cells across all tested DOX concentrations (Figure 4E,F). Co-incubation of DOX (1 µM) with SFN (1 µM) increased the cells’ ability to retain NR, from 82.53 ± 3.32% to 94.55 ± 4.13%, and improved MTT reduction, from 84.11 ± 3.46% to 88.62 ± 3.50%. For DOX at 10 µM, SFN at 1 µM increased values from 63.90 ± 3.70% to 87.34 ± 4.36% in the NR uptake assay and from 61.30 ± 3.92% to 69.20 ± 3.71% in the MTT reduction assay.

Lastly, cheirolin (CH) protected SH-SY5Y cells against DOX-induced toxicity. In the NR uptake assay, CH protected SH-SY5Y cells against the toxicity caused by DOX 1 µM (83.17 ± 3.43% to 93.24 ± 4.92%) and by DOX 10 µM (69.02 ± 4.37% to 80.90 ± 5.34%) (Figure 4G). In the MTT reduction assay, however, the co-incubation of DOX 1 µM with CH did not show any protective ability. However, CH conferred partial protection to differentiated SH-SY5Y against DOX 10 µM-induced toxicity (63.40 ± 4.71% to 70.42 ± 8.46%) (Figure 4H).

### 2.4. N-Acetyl Cysteine, Dimethyl Fumarate, Sulphoraphane, and Cheirolin Were Partially Protective Against Sunitinib’s Cytotoxicity

**Figure 5:**
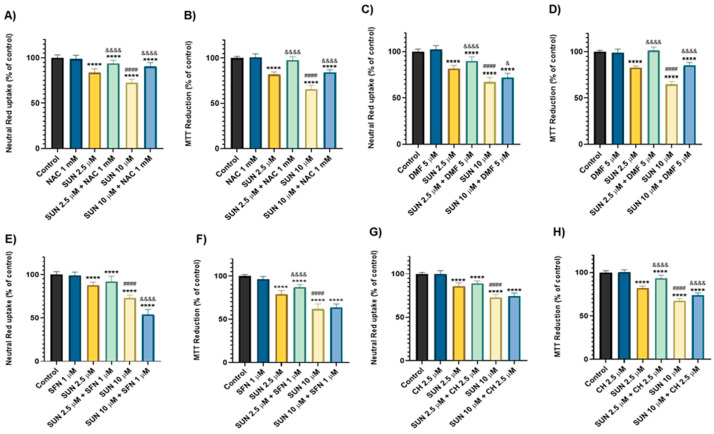
NR uptake and MTT reduction assays in differentiated SH-SY5Y cells exposed to 2.5 and 10 μM of SUN and NAC 1 mM (**A**,**B**), DMF 5 µM (**C**,**D**), SFN 1 µM (**E**,**F**), and CH 2.5 µM (**G**,**H**) for 24 h. Data are presented as mean ± SD from four independent experiments (N = 16). Statistical analysis was conducted using a one-way ANOVA, followed by the Tukey’s post hoc test (**** *p* < 0.0001 vs. control; ^####^ *p* < 0.0001 vs. the lower concentration of the anticancer drug; ^&^ *p* < 0.05, ^&&&&^ *p* < 0.0001 vs. the same concentration without the redox homeostasis modulator).

The co-incubation with SUN and NAC was partially protective to differentiated SH-SY5Y cells. In the lowest concentration of SUN (2.5 µM), NAC offered protection in both the NR uptake assay (83.72 ± 4.21% to 93.98 ± 3.27%) and the MTT reduction assay (81.91 ± 2.86% to 97.88 ± 3.64%) (Figure 5A,B). For the highest concentration of SUN, similar results were observed with NAC, which conferred protection in the NR uptake assay (72.73 ± 3.75% to 90.48 ± 4.13%) and the MTT reduction assay (65.67 ± 4.28% to 84.31 ± 2.87%) (Figure 5A,B).

As for the co-incubation of SUN with DMF 5 μM, this modulator protected differentiated SH-SY5Y cells against SUN’s toxicity. In the NR uptake assay, the incubation with DMF 5 µM improved cells’ ability to uptake the vital dye when cells were exposed to SUN 2.5 µM (81.60 ± 3.45% to 89.87 ± 4.53%) and SUN 10 µM (67.26 ± 4.97% to 72.05 ± 4.49%) (Figure 5C). In the MTT reduction assay, DMF 5 µM also conferred protection against SUN 2.5 µM (82.84 ± 1.90% to 101.3 ± 3.88%) and SUN 10 µM (64.89 ± 3.03% to 85.30 ± 3.18%) induced cytotoxicity (Figure 5D). DMF alone did not induce significant changes in either assay (NR: 102.4 ± 4.15% and MTT: 99.00 ± 3.86%) compared to the control (NR: 100 ± 2.80% and MTT: 100 ± 1.57%) (Figure 5C,D).

SFN at 1 μM exhibited differential effects on SUN-induced cytotoxicity, depending on the anticancer drug’s concentration. The NR uptake assay showed that SFN 1 μM increased the toxicity of SUN 10 μM, from 53.94 ± 5.94% in the co-incubation with SFN compared to 72.98 ± 3.40% with SUN 10 μM alone (Figure 5E). However, SFN 1 μM did not significantly alter the toxicity at the lower concentration of SUN 1 μM. In contrast, the MTT reduction assay indicated that SFN 1 μM protected differentiated SH-SY5Y cells against SUN at 2.5 μM, with values shifting from 79.00 ± 4.12% to 86.96 ± 3.16% (Figure 5F). For SUN 10 μM and SFN, no changes were seen in the MTT reduction assay (Figure 5F). SFN alone did not cause significant cytotoxicity in either test performed (NR: 98.96 ± 3.90% and MTT: 96.49 ± 3.14%) compared to the respective controls.

The NR uptake assay showed that CH did not provide any protection against either of the tested concentrations of SUN (Figure 5G). However, in the MTT reduction assay, CH provided protection against both concentrations of SUN. Co-incubation with CH increased MTT reduction ability from 82.13 ± 2.34% to 93.42 ± 3.51% at SUN 2.5 μM, and from 67.29 ± 2.60% to 72.72 ± 2.72% at SUN 10 μM (Figure 5H). CH did not cause any significant changes, per se (NR: 99.62 ± 4.16% and MTT: 100.4 ± 2.80%), compared to the control (NR: 100 ± 1.60% and MTT: 100 ± 2.14%).

### 2.5. Autophagy Seems to Play a Key Role in Sunitinib’s Toxicity

As observed in the morphology analysis, cells exposed to SUN displayed yellow inclusions, which were more prominent at higher concentrations (5 and 10 μM). To evaluate the role of autophagy in SUN’s toxicity, cells were exposed to three different autophagy modulators: 3-methyladenine (3-MA), chloroquine (CLQ), and rapamycin (RAP).

**Figure 6:**
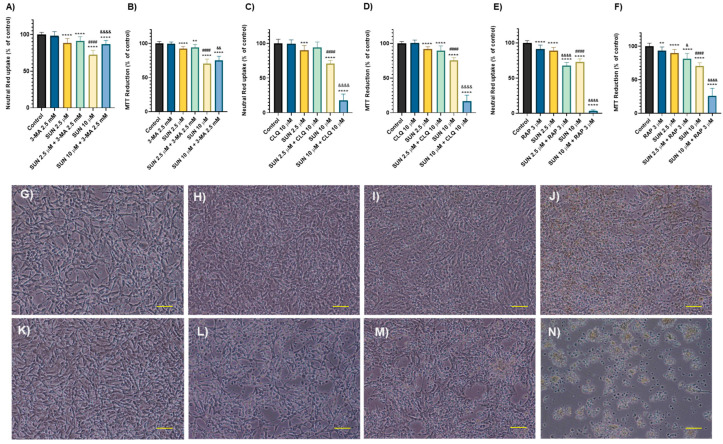
NR uptake and MTT reduction assays in differentiated SH-SY5Y cells exposed to 2.5 and 10 μM of SUN and 3-MA 1 mM (**A**,**B**), CLQ 10 µM (**C**,**D**) and RAP 3 µM (**E**,**F**) for 24 h. Data are presented as mean ± SD from 3–5 independent experiments (N = 15 to 20). Statistical analysis was conducted using a one-way ANOVA, followed by the Tukey’s post hoc test (** *p* < 0.01, *** *p* < 0.001, **** *p* < 0.0001 vs. control; ^####^ *p* < 0.0001 vs. the lower concentration of the anticancer drug; ^&^ *p* < 0.05, ^&&^ *p* < 0.01, ^&&&&^ *p* < 0.0001 vs. the same concentration without the autophagy modulator). Morphological analysis by phase contrast microscopy of differentiated SH-SY5Y cells after a 24 h exposure to SUN 2.5 μM and 3-MA 1 mM (**I**), SUN 10 μM and 3-MA 1 mM (**J**), SUN 2.5 μM and CLQ 10 μM (**M**) and SUN 10 μM and CLQ 10 μM (**N**), using medium (**G**,**K**), and 3-MA 1 mM (**H**) and CLQ 10 μM (**L**) as controls. Representative images of two independent experiments. Scale bare: 100 µm.

3-MA protected SH-SY5Y cells from the cytotoxicity induced by the highest concentration of SUN. Specifically, 3-MA decreased the cytotoxicity in SUN 10 μM-treated cells, as measured by both the NR uptake assay (from 72.38 ± 6.09% to 87.06 ± 4.97%) and the MTT reduction assay (from 70.13 ± 6.88% to 75.27 ± 5.92%). However, at the lower concentration of SUN (2.5 μM), no significant changes in cytotoxicity were observed in either assay (Figure 6A,B). Of note, 3-MA alone did not induce significant changes (NR: 98.19 ± 5.81% and MTT: 99.57 ± 2.58%) when compared to the controls (NR: 100 ± 2.98% and MTT: 100 ± 2.84%).

CLQ enhanced the cytotoxicity of SUN at the highest concentration. At the lowest tested concentration of SUN (2.5 μM), CLQ did not induce any significant changes compared to SUN alone at the same concentration (Figure 6C,D). However, in the highest concentration (SUN 10 μM), CLQ significantly increased the cytotoxicity of SUN in the NR uptake assay (70.53 ± 4.90% to 17.95 ± 9.02%) and the MTT reduction assay (75.65 ± 4.01% to 16.57 ± 8.76%) (Figure 6C,D). CLQ alone did not lead to significant changes (NR: 99.10 ± 5.95% and MTT: 100.7 ± 4.06%) when compared to the control cells (NR: 100 ± 5.91% and MTT: 100 ± 2.82%).

RAP increased the cytotoxicity at both SUN concentrations (2.5 and 10 µM) (Figure 6E,F). In the case of the lowest concentration of SUN, co-incubation with RAP increased the toxicity of SUN in both NR uptake (from 88.90 ± 4.50% to 67.48 ± 4.55%) and the MTT reduction assay (from 89.77 ± 5.43% to 81.30 ± 7.80%). For the highest concentration of SUN (10 µM), RAP caused a substantial increase in SUN’s toxicity, as shown by the NR uptake assay (from 72.84 ± 4.32% to 3.47 ± 1.29%) and the MTT reduction assay (from 70.47 ± 4.96% to 25.66 ± 11.55%) (Figure 6F). However, it cannot be conclusively stated that RAP increases the toxicity of SUN, as RAP is inherently cytotoxic (NR: 91.32 ± 5.58% and MTT: 93.23 ± 5.61%).

Regarding the morphological analysis, differentiated SH-SY5Y cells co-incubated with 3-MA and SUN presented slight morphological alterations, including some rounding of the cell’s body (Figure 6I,J), when compared to the control group (Figure 6G). Notably, cells exposed to both SUN and CLQ showed more visible changes in their morphology (Figure 6M,N) compared to the control group (Figure 6K). The effects were more pronounced at SUN 10 µM with CLQ, where a synergistic increase in toxicity was evident, leading to extensive cell rounding and detachment (Figure 6N).

**Figure 7:**
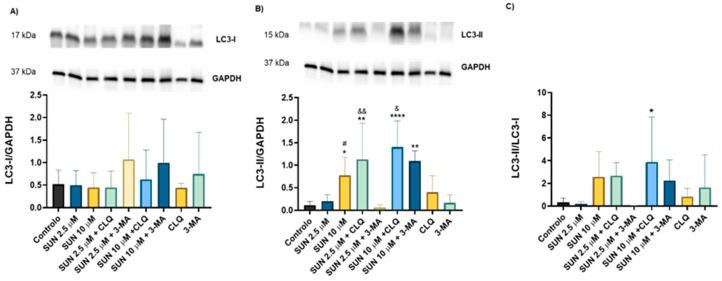
Expression of LC3 form I (17 kDa) (**A**) or LC3 form II (15 kDa) (**B**) when assessed by western blotting after SUN (2.5 µM and 10 µM) treatment with or without the autophagy modulators CLQ (10 µM) and 3-MA (2.5 mM). Data are presented as mean ± SD from 3–4 independent experiments of the ratio between optic density of LC3-I (17 kDa) (or LC3-II, 15 kDa) and the loading control (GAPDH, 37 kDa). A representative image of the blot is provided for all the conditions. The ratio between arbitrary optic density units of LC3 subunit II (15 kDa) and LC3 subunit I (17 kDa) determined by western blotting after SUN (2.5 µM and 10 µM) treatment with or without the autophagy modulators CLQ (10 µM) and 3-MA (2.5 mM) are represented on the bar charts (**C**). Statistical comparisons were made using a one-way ANOVA, followed by Fisher’s LSD post hoc test (* *p* < 0.05, ** *p* < 0.01, **** *p* < 0.0001 vs. control; ^#^ *p* < 0.05 vs. the lower concentration of the anticancer drug; ^&^ *p* < 0.05, ^&&^ *p* < 0.01 vs. the same concentration without the autophagy modulator).

The morphological results, along with the findings from the autophagy modulators used, suggest that autophagy may be involved in SUN’s toxicity. To explore this further, the levels of LC3-I and LC3-II, well-established markers of autophagy, were quantified. Western blot analysis showed no significant changes in LC3-I levels under any tested condition (Figure 7A). However, differences were observed in the levels of LC3-II (Figure 7B). Compared to the control group, neither autophagy modulator used induced significant changes in LC3-II levels. Cells exposed to SUN at 2.5 µM also showed no significant alterations, but exposure to SUN at 10 µM resulted in a significant increase in LC3-II levels. Co-incubation of SH-SY5Y cells with CLQ led to increased LC3-II levels at both SUN concentrations. Co-incubation with SUN at 10 µM and 3-MA also increased LC3-II levels, while 3-MA did not affect LC3-II levels in cells treated with SUN at 2.5 µM (Figure 7B). The LC3-II/LC3-I ratio showed significant differences only in cells co-incubated with SUN at 10 µM and CLQ, with no significant differences in other conditions compared to the control group (Figure 7C).

## 3. Discussion

Chemotherapy-induced cognitive impairment, commonly known as “chemobrain”, significantly impacts cancer survivors, affecting memory, attention, and processing speed. While its exact mechanisms remain unclear, growing evidence points to the involvement of oxidative stress, mitochondrial dysfunction, neuroinflammation, and autophagy dysregulation. While most research has been focused on traditional chemotherapeutic agents like DOX, the potential neurotoxic effects of newer targeted therapies, such as SUN, remain largely unexplored. Understanding how these drugs contribute to chemobrain is essential for developing neuroprotective strategies and improving the quality of life for cancer patients or survivors. Thus, the main objective of this work was to compare the neurotoxicity of DOX and SUN and assess their involvement in the development of chemobrain, using human differentiated SH-SY5Y cells.

Dopamine (DA) plays a central and complex role in various cognitive functions [40]. It is synthesized and released by dopaminergic neurons originating primarily from the ventral tegmental area (VTA) and the substantia nigra. These neurons project to several brain regions, including the nucleus accumbens, amygdala, medial prefrontal cortex, striatum, and hippocampus [40]. Growing evidence suggests that chemotherapy-induced cognitive impairment may be associated with disruptions in dopaminergic signaling. Vitor et al. reported that patients with cognitive impairment following cancer treatment exhibit significant alterations in DAT and a notable dopaminergic decrease in the dorsal striatum compared to healthy controls [41]. Similarly, Kaplan and colleagues observed decreased levels of DA in the brain of carboplatin-treated rats, further supporting the involvement of DA dysregulation in chemobrain [42]. DOX has been shown to exert a biphasic effect on DA release, as demonstrated in a study using Wistar rats [43]. Short-term DOX exposure appeared to enhance DA system activity and increase impulsivity, while prolonged exposure led to DA suppression, potentially contributing to cognitive deficits [43]. In contrast, no studies have specifically examined DA levels following SUN administration. Thus, to better understand the impact of DOX and SUN on dopaminergic neurons, SH-SY5Y cells were differentiated using retinoic acid (RA) and 12-O-tetradecanoylphorbol-13-acetate (TPA), as previously described by our group [44], and then exposed to both drugs. This differentiation method promotes the dopaminergic phenotype and reduces the mitotic rate while enhancing the dopaminergic phenotype, as mentioned in [44,45,46]. As mentioned, the concentrations used for both drugs were based on clinically relevant levels found in the plasma and brain of treated patients [12,38,39].

Both DOX and SUN induced significant cytotoxicity in differentiated SH-SY5Y cells in a time- and dose-dependent manner. At lower concentrations, DOX at 0.1 µM did not induce significant cytotoxicity in either MTT reduction or NR uptake assays, which is consistent with previous reports showing minimal effects at this concentration [47]. However, at the higher concentrations of 1 and 10 µM, cytotoxicity increased markedly, particularly at 48 h, aligning with findings from other studies [48,49]. SUN at 1 µM showed early toxicity in the MTT assay, suggesting potential mitochondrial stress. Interestingly, NR uptake did not show significant cytotoxicity at this concentration, likely due to differences in the sensitivity of the assays. This suggests that SUN at 1 µM induces mitochondrial dysfunction without immediately impacting lysosomal integrity. At higher concentrations (1–10 µM for DOX; 2.5–10 µM for SUN), both drugs significantly exacerbated cytotoxicity at 48 h, as confirmed by both assays. While DOX’s cytotoxicity at these doses aligns with its well-documented off-target neurotoxicity [48,49], SUN’s neurotoxic potential was less anticipated due to its targeted nature as a multi-tyrosine kinase inhibitor, being this the first in vitro study that has ever been conducted on a neuronal model.

Given that DOX has been shown to cause mitochondrial damage [15,50] and that SUN exhibited early toxicity in the MTT reduction assay, a comparative analysis was conducted to assess changes in mitochondrial membrane potential. The results demonstrated a concentration-dependent reduction in Δψm for both DOX and SUN. Interestingly, while DOX 0.1 µM did not induce significant cytotoxicity at 24 h in both cytotoxicity assays, it caused meaningful mitochondrial membrane depolarization. This aligns with previous findings in non-differentiated SH-SY5Y cells, where DOX 0.13 µM led to evident Δψm depolarization using a DiO6 mitochondrial probe [48]. Similarly, Ongnok and colleagues reported mitochondrial membrane depolarization in the brains of DOX-treated rats (3 mg/kg, i.p.), which was accompanied by increased oxidative stress [11].

For SUN, significant mitochondrial membrane depolarization was observed at 5 µM and 10 µM, whereas 2.5 µM did not induce detectable Δψm changes despite its significant cytotoxic effect in the MTT assay. This suggests that SUN at 2.5 µM may impair cellular metabolism without immediately disrupting mitochondrial potential, though further studies are needed to elucidate the underlying mechanisms involved. Despite the limited research on SUN’s neurotoxicity and its role in chemobrain, existing studies on its adverse effects in other organs suggest that it causes mitochondrial impairments [51]. These findings suggest that DOX may cause early mitochondrial dysfunction at sub-cytotoxic concentrations, while SUN induces metabolic stress and cytotoxicity at higher doses without immediate disruption of mitochondrial potential.

The presence of ABC transporters in these cells cannot be overlooked, as both SUN and DOX are known substrates of these efflux proteins. Consequently, their potential impact on the observed cellular responses must be considered. Although the available literature on MDR1 expression following SH-SY5Y cell differentiation with RA and TPA is limited, we cannot exclude the possibility that such expression may influence the intracellular accumulation of both drugs in our model, and the overall cytotoxicity observed [28,52,53,54]. Oxidative stress has been identified as a key factor in chemobrain pathology and is closely linked to inflammation. Oxidative damage promotes TNF-α production in peripheral tissues, which crosses the BBB and triggers further oxidative and nitrosative stress, mitochondrial dysfunction, and neuronal death [14]. TNF-α also activates NF-κB, a transcription factor that induces pro-inflammatory genes and has been shown to increase following chemotherapy, particularly with DOX [55,56]. Factor nuclear kappa B (NF-κB) and nuclear factor (erythroid-derived 2)-related-factor 2 (Nrf2), two critical regulators of oxidative stress and inflammation, interact antagonistically: NF-κB suppresses Nrf2 activity, while Nrf2 can inhibit NF-κB expression [57]. Nrf2 plays a key role in antioxidant defense by regulating genes such as HO-1, NQO1, and the modulatory and catalytic subunits of γ-glutamyl cysteine ligase (GCLM and GCLC, respectively, which are responsible for GSH synthesis), as well as ferritin [58]. Under normal conditions, Keap1 targets Nrf2 for degradation, but several intracellular processes, mainly related to oxidative stress, disrupt this process, allowing Nrf2 to release itself and accumulate in the nucleus, act on the antioxidant response elements (AREs), and activate antioxidant pathways [58]. While NF-κB has been extensively studied in chemobrain, the role of Nrf2 remains less explored. Given its potential in mitigating oxidative stress, this study examined four Nrf2-associated redox modulators—NAC, DMF, SFN, and CH—to assess their impact on anticancer drug toxicity.

In this study, NAC’s role in modulating the cytotoxicity of DOX and SUN revealed distinct outcomes, highlighting the context-dependent nature of its effects. When SH-SY5Y cells were co-incubated with NAC and DOX (1 µM), the NR uptake assay indicated an increase in cytotoxicity. This aligns with findings in human ovarian cancer cells (CaOV3), where NAC enhanced DOX-induced ATM and p53 phosphorylation, potentiating its effects [59]. However, at a higher DOX concentration (10 µM), NAC did not significantly alter toxicity, possibly because the dominant cytotoxic mechanisms of DOX overshadowed NAC’s modulatory influence. Notably, the MTT reduction assay did not reveal significant differences between DOX-treated and DOX-NAC co-treated cells, suggesting that NAC’s impact in the NR assay may be linked to specific cellular processes, such as lysosomal integrity, rather than overall metabolic activity. Consistent with these findings, Almeida et al. reported that NAC (1 mM) did not prevent DOX-induced cytotoxicity in non-differentiated SH-SY5Y cells [48]. Nonetheless, several in vitro and in vivo studies suggest that NAC offers neuroprotection against DOX-induced toxicity, particularly by preserving GSH levels and mitigating histopathological damage in animal models and cells [60,61,62].

Conversely, NAC displayed a protective effect against SUN-induced toxicity in differentiated SH-SY5Y cells. These results are consistent with previous studies in non-neuronal models, where NAC reduced ROS levels and prevented SUN-induced mitochondrial apoptosis in L02 cells (human hepatocytes) and Caki-1 and 786-O cells (human renal cell carcinoma models) [51,63]. However, no prior studies have specifically examined NAC’s interaction with SUN in neuronal cells.

Co-incubation with DMF significantly reduced DOX (1 µM)-induced cytotoxicity in both the NR uptake and MTT reduction assays, which is consistent with studies showing DMF’s protective role through Nrf2 activation and antioxidant defense enhancement [64,65]. However, at a higher DOX concentration (10 µM), DMF exacerbated its toxicity. This paradoxical effect may be due to an overload of oxidative stress at higher DOX concentrations, overwhelming the protective capacity of DMF or acting on different mechanisms. As dual cellular responses towards DOX neurotoxicity on cortical neurons have been found [49], one could also hypothesize that DMF may have different abilities to counteract dissimilar cellular responses. Conversely, DMF conferred consistent protection against SUN toxicity at both tested concentrations. DMF significantly reduced the cytotoxicity of SUN at 2.5 µM, as well as at 10 µM, although to a lesser extent in the latter condition. Given SUN’s well-documented role in inducing oxidative stress on other organs [36,37], DMF’s effect is likely mediated by Nrf2 activation.

As for SFN, the results obtained in this study indicate that SFN (1 µM) provides significant protection against DOX-induced cytotoxicity in differentiated SH-SY5Y cells. Both NR uptake and MTT reduction assays confirmed that SFN reduced DOX toxicity across all tested concentrations, including at 10 µM, where DMF instead exacerbated cytotoxicity. SFN’s protective effects align with studies demonstrating its role in preventing DOX-induced apoptosis and ROS formation via Nrf2 activation [66,67,68]. Notably, despite both SFN and DMF being Nrf2 activators, their effects on DOX diverge. DMF’s broader interactions with protein thiols [69], and its relatively weaker antioxidant and anti-inflammatory properties compared to SFN [70], may explain this discrepancy. Surprisingly, regarding SUN, SFN displayed a concentration-dependent effect. At 2.5 µM SUN, SFN provided protection in the MTT reduction assay, but at 10 µM, it exacerbated toxicity in the NR uptake assay, suggesting a context-dependent response.

The final redox homeostasis modulator tested, CH (an analogue of SFN), protected differentiated SH-SY5Y cells from DOX-induced cytotoxicity, as shown by NR uptake and MTT reduction assays. At 10 µM DOX, CH significantly reduced cytotoxicity, while at 1 µM DOX, protection was observed in the NR uptake assay, but not in the MTT reduction assay. No studies have yet explored CH’s protective role against DOX toxicity. For SUN, CH provided partial protection in SH-SY5Y cells; while no significant effects were observed in the NR uptake assay, the MTT reduction assay showed modest protection. These differences highlight the distinct effects of redox homeostasis modulators in mitigating the neurotoxicity induced by DOX and SUN in differentiated SH-SY5Y cells. DOX-induced toxicity was modulated in a concentration-dependent manner, with some modulators (NAC and DMF) exacerbating toxicity at higher DOX concentrations, while others (SFN and CH) provided consistent protection. This suggests that DOX toxicity may involve complex redox interactions beyond oxidative stress alone. Conversely, SUN-induced toxicity was more consistently attenuated by redox modulators, with NAC, DMF, SFN, and CH all offering some degree of protection. These results highlight the potential of redox homeostasis modulators in protecting against chemobrain, though their efficacy depends on the specific chemotherapeutic agent and its concentration.

On the other hand, autophagy is a fundamental cellular process responsible for degrading long-lived proteins, protein aggregates, and damaged organelles to maintain cellular homeostasis [71]. Abdel-Aziz and colleagues reported impairments in the autophagic machinery in SUN-treated mice, evidenced by decreased levels of beclin-1 and Atg5, alongside an accumulation of p62/SQTM1 in the cortex and hippocampus [34]. These alterations were linked to cognitive impairment [34].

The results obtained in SH-5YSY from the morphology analysis revealed that cells exposed to SUN presented yellow inclusions, particularly at the highest concentrations (5 and 10 μM). These morphological alterations suggest a potential disturbance in cellular homeostasis. The accumulation of yellow inclusions may indicate a disruption in autophagic processes, which is supported by the existing literature and therefore warrants further investigation into the role of autophagy in SUN-induced toxicity. To investigate the role of autophagy in SUN-induced toxicity, differentiated SH-SY5Y cells were co-incubated with autophagy modulators: 3-MA, CLQ, and RAP. 3-MA is a widely used inhibitor of autophagy that targets the class III phosphatidylinositol 3-kinase (PI3K), thereby preventing the initiation of autophagosome formation [72,73]. In the present study, 3-MA conferred protection towards the cytotoxicity induced by SUN, particularly against the highest SUN concentration, suggesting that early-stage autophagy contributes to SUN-induced cytotoxicity. Excessive or prolonged autophagy can promote cell death through excessive self-digestion and degradation of essential cellular components [74]. By inhibiting autophagy initiation, 3-MA may alleviate cellular stress from dysregulated autophagic activity, enhancing cell survival. These findings suggest that under severe stress, such as high SUN concentrations, autophagy may shift from a protective to a detrimental role, contributing to cell death.

The second autophagy modulator tested was CLQ, which inhibits autophagy by disrupting lysosomal acidification and preventing autophagosome-lysosome fusion, leading to autophagic vacuole accumulation [72,75]. Co-treatment of SH-SY5Y cells with SUN and CLQ significantly exacerbated SUN-induced cytotoxicity, particularly at 10 µM. By blocking autolysosome formation, CLQ may impair the clearance of damaged organelles and proteins, contributing to cell death [76]. This disruption could underline cognitive deficits associated with autophagy impairment, as observed in mice showing synaptic protein accumulation and cognitive decline following autophagy inhibition [77]. These findings suggest that functional lysosomal activity and autophagosome fusion are critical for protecting neurons against SUN-induced toxicity. As a weak base (pKa ~8.95), SUN accumulates in lysosomes through pH-dependent trapping, becoming protonated and sequestered within acidic organelles. SUN has been shown to neutralize lysosomal pH (to approximately pH 7.5), thereby inhibiting cathepsin B activity and disrupting autophagic flux. This disruption results in incomplete autophagy, characterized by the accumulation of undegraded autolysosomes, as observed in our phase-contrast microscopy analysis. CLQ is a lysosomotropic agent that elevates lysosomal pH, inhibiting both autophagosome–lysosome fusion and the activity of lysosomal degradative enzymes (e.g., cathepsins). CLQ may exacerbate SUN-induced lysosomal dysfunction by further alkalinizing lysosomes, thereby amplifying autolysosome accumulation and overwhelming lysosomal processing capacity. Both SUN and CLQ impair lysosomal function and autophagy, contributing to enhanced cellular toxicity through overlapping and potentially synergistic cytotoxic mechanisms [54,78].

The last autophagy modulator tested was RAP, which is an autophagy inducer that inhibits mTOR, thereby promoting autophagy [79,80]. Co-incubation of RAP with SUN (2.5 µM) significantly increased toxicity, though this effect may partly arise from RAP’s own cytotoxicity. At 10 µM SUN, RAP dramatically potentiated cytotoxicity, suggesting a strong synergistic interaction.

To further investigate the role of autophagy in SUN-induced neurotoxicity, LC3 levels were quantified by western blotting. In this study, LC3-I levels remained unchanged across conditions, suggesting that basal autophagy in differentiated SH-SY5Y cells was not significantly affected by SUN or autophagy modulators. However, LC3-II levels increased significantly at 10 µM SUN, indicating enhanced autophagic activity and potential autophagosome accumulation. The effects of autophagy modulators could help to further clarify its role on the highest SUN concentration. CLQ, which inhibits lysosomal function, significantly increased LC3-II levels in SUN-treated cells, confirming that blocking autophagosome degradation leads to their accumulation. Previous studies in OS-RC-2 cells have reported similar results, with CLQ enhancing SUN’s cytotoxicity and increasing LC3, Atg5, and Beclin-1 levels [75]. Furthermore, CLQ augmented apoptotic cell death, supporting the hypothesis that impaired autophagic flux exacerbates toxicity [75]. In contrast, 3-MA, an inhibitor of autophagosome formation, displayed a more complex interaction. Co-treatment with 3-MA and 10 µM SUN led to increased LC3-II levels, suggesting autophagosome accumulation due to early-stage inhibition. This protective effect may arise from preventing cells from engaging in a dysfunctional autophagic response. Consistent with this, 3-MA was shown to reduce neuronal death and cognitive deficits in spinal cord ischemia/reperfusion models [74]. At 2.5 µM SUN, 3-MA had no significant effect on LC3-II levels, suggesting that lower SUN concentrations induce lower or none autophagic stress. Although we did not observe substantial morphological changes indicative of autophagic disruption in our model with DOX, we cannot disregard its known effects. DOX is reported to inhibit lysosomal acidification, thereby blocking autophagic flux and leading to the accumulation of toxic autolysosomes in other cellular models [81].

## 4. Materials and Methods

### 4.1. Materials

Trypsin-EDTA, dimethyl sulfoxide (DMSO), NR solution, 3-(4,5-dimethylthiazol-2-yl)-2,5-diphenyltetrazolium bromide (MTT), TPA, RA, NAC, d,l-sulforaphane (SFN), DMF, RAP, CLQ, LC3 antibody (L7543), phenylmethanesulfonylfluoride fluoride, sodium dodecyl sulphate (SDS), and the protease inhibitor cocktail (P8340) were obtained from Sigma-Aldrich (St. Louis, MO, USA). Dulbecco’s Modified Eagle’s Medium (DMEM) high glucose was obtained from Gibco (New York, NY, USA), while heat-inactivated fetal bovine serum (FBS), penicillin/streptomycin (10,000 U/mL; 10,000 μg/mL), and Hank’s Balanced Salt Solution (HBSS) with calcium and magnesium were obtained from Pan Biotech (Aidenbach, Germany). Doxorubicin hydrochloride (ab120629), CH, 3-MA, and antibodies, including anti-mouse (ab6728) and anti-rabbit (ab97051), were obtained from Abcam (Cambridge, UK). Sunitinib and the GAPDH antibody (SC47724) were obtained from Santa Cruz Biotechnology (Dallas, TX, USA). Materials for western blotting and protein analysis, including TRIS/Glycine, the DCTM Protein Assay Kit (Cat #5000112), Precision Plus Protein Dual Color Standards (Cat #1610374), Mini-Protean TGX stain-free gels, Trans-Blot Turbo Transfer Packs, 4× Laemmli Sample Buffer, and Clarity™ Western ECL substrate (Cat #170-5061), were obtained from BIO-RAD Laboratories, Inc. (Hercules, CA, USA). Dulbecco’s phosphate-buffered saline (PBS) without calcium and magnesium was obtained from Biochrom (Berlin, Germany). Cell culture pipettes were obtained from Nerbe Plus (Winsen, Germany), while 25 cm^2^ culture flasks were obtained from Corning (New York, NY, USA), and 48- and 6-well plates were obtained from TPP (Trasadingen, Switzerland).

### 4.2. Cell Culture

In this study, the human neuroblastoma cell line SH-SY5Y was used, being obtained from Sigma-Aldrich (St. Louis, MO, USA). In the experimental setup, SH-SY5Y cells were maintained in DMEM supplemented with 10% FBS and 1% penicillin/streptomycin (10,000 U/mL and 10,000 μg/mL, respectively) in 25 cm^2^ cell culture flasks at 37 °C under a 5% CO_2_ atmosphere. Upon reaching approximately 80–90% confluence, cells were harvested by trypsinization and subcultured at a density of 25,000 cells/cm^2^. Cell density was determined using a Neubauer chamber and the trypan blue exclusion assay. Specifically, 48-well plates were utilized for cytotoxicity assays, 24-well plates for morphological analysis, and 6-well plates for cell retrieval for protein analysis.

### 4.3. Cell Differentiation

DA, synthesized and released by dopaminergic neurons, plays a central and complex role in various cognitive functions [40]. Studies have reported alterations in DA and dopamine transporter (DAT) in the brain of patients experiencing cognitive impairment after cancer treatment [41] and carboplatin-treated rats [42]. Thus, dopaminergic neuronal cells represent an appropriate model for studying chemobrain. To promote a dopaminergic phenotype, SH-SY5Y cells were treated with RA and TPA, following previous works performed in our laboratory [44,46,48]. This differentiation method leads to accentuated morphological changes (extension of neurites and more elongated shape), significant increases in the expression of DAT and tyrosine hydroxylase (TH), and an increased capacity to accumulate DA, as well as higher resistance against DA-induced neurotoxicity on the cells [44,46]. To start the differentiation, cells were seeded at a density of 25,000 cells/cm^2^ in complete DMEM medium supplemented with 10 µM RA for three days. On the third day, the medium was replaced with complete DMEM containing 80 µM TPA, and the cells were maintained for an additional three days.

### 4.4. Drug Exposure

After the differentiation process, the cells were exposed to clinically relevant doses of DOX (0.1, 1, and 10 µM) and SUN (1, 2.5, 5, and 10 µM) for 24 or 48 h. DOX was dissolved in sterile phosphate buffer, while SUN stock solutions were prepared in DMSO. DMSO was also tested as a vehicle in SUN’s experiments. After the exposure period, NR uptake and MTT reduction assays, morphological analysis, and mitochondrial membrane potential (Δψm) evaluation were conducted. To explore potential protectors against the cytotoxicity induced by DOX and SUN, differentiated SH-SY5Y cells were incubated alongside 4 different redox homeostasis modulators—NAC (1 mM), DMF (5 µM), SFN (1 µM), and CH (2.5 µM)—related to the Nrf2 pathway. All samples were co-incubated with DOX or SUN, except for DMF, which was pre-incubated for 90 min before cells’ exposure to anticancer drugs.

Under basal conditions, Nrf2 is sequestered in the cytoplasm by Kelch-like ECH-associated protein 1 (Keap1), which facilitates its ubiquitination and degradation via the proteasome [82]. However, under oxidative or electrophilic stress, Keap1 undergoes conformational changes, leading to the release of Nrf2. Once released, Nrf2 moves to the nucleus, where it binds to antioxidant response elements (AREs) in the promoter regions of target genes [82]. This triggers the expression of antioxidant genes, including heme oxygenase-1 (HO-1) and NAD(P)H dehydrogenase quinone 1 (NQO1) [58].

NAC is a GSH precursor. After uptake into the cells, it is hydrolyzed to cysteine and incorporated into GSH, the most abundant cellular antioxidant [83]. By increasing the intracellular levels of GSH, NAC helps to neutralize reactive oxygen species (ROS) and reduce oxidative stress [83]. Several studies linked NAC with Nrf2. Some studies report that NAC is an activator of the Nrf2 pathway [84,85,86]. However, some studies suggest that NAC inhibits this pathway [82]. Furthermore, while it is commonly assumed that all thiols possess the antioxidant capacity of GSH, NAC does not effectively scavenge one of the most abundant radical species, the O_2_^•−^ [87]. However, it is a potent scavenger of hypochlorous acid, reacts with HO^•^, and reacts with H_2_O_2_ [87].

DMF is an activator of the Nrf2 pathway [65,88]. DMF oxidizes the sulfhydryl (-SH) groups on Keap1, causing Keap1 to dissociate from Nrf2 [89]. This allows Nrf2 to migrate to the nucleus, where it promotes the expression of genes involved in antioxidant defenses [89].

SFN is an isothiocyanate present in cruciferous vegetables (broccoli and kale) in its inactive precursor, glucoraphanin [90]. Glucoraphanin is converted into SFN by the enzyme myrosinase, which is released when plant tissues are damaged (e.g., by chewing or cutting them) [90]. SFN is an activator of the Nrf2 pathway, stimulating the expression of genes associated with antioxidant functions [90,91].

CH is an analogue of SFN, with a similar capacity to SFN in activating Nrf2 dependent gene expression [92]. However, a study performed by Ernst and colleagues suggested that CH may also induce Nrf2 through an extracellular signal-related kinase (ERK)-dependent signal-transduction pathway [93].

The role of autophagy in SUN’s toxicity was also evaluated, using different autophagy modulators: 3-MA (2.5 mM), CLQ (10 µM) and RAP (3 µM). 3-MA, an autophagy inhibitor, targets the early stage of autophagy by inhibiting class III phosphatidylinositol 3-kinase, a key mediator of vesicle nucleation [72,73]. CLQ, another autophagy inhibitor, disrupts the autophagic process by preventing autophagosome-lysosome fusion, resulting in the accumulation of autophagic vacuoles [73,75]. RAP (an autophagy inducer), on the other hand, triggers the autophagic process by inhibiting the mechanistic target of rapamycin complex 1 (mTORC1) [80]. Activated mTOR inhibits autophagy; therefore, by inhibiting the mTOR complex, RAP promotes autophagy [79].

### 4.5. Cytotoxicity and Morphological Analysis

#### 4.5.1. Cellular Morphology

The morphology of the differentiated SH-SY5Y cells exposed to DOX or SUN for 24 and 48 h was evaluated using phase contrast microscopy. Imaging was performed with a Nikon Eclipse TS100 microscope (Tokyo, Japan) equipped with a Nikon DS-Fi1 camera (Tokyo, Japan).

#### 4.5.2. The Neutral Red Uptake Assay

The NR uptake assay relies on the ability of viable, intact cells to bind to the supravital dye NR, which accumulates within the lysosomes [94,95]. After the exposure period, the cells’ medium was removed from each well and warm medium with NR (33 µg/mL) was added. The cells were maintained at 37 °C for 90 min, protected from light. Then, the medium was aspirated, and the cells were washed with warm HBSS containing calcium and magnesium. The HBSS was removed from each well, and ethanol/acetic acid solution (1 mL acetic acid, 50 mL 100% ethanol and 49 mL distilled water) was added. The plates were protected from the light and were placed in a shaker for about 15 min. Finally, the absorbance was read at 540 nm and 690 nm (reference wavelength) in a Biotek Synergy HT microplate reader (Winooski, VT, USA), as described in [48]. The results were expressed as a percentage relative to the control (set to 100%) after subtracting the values of the reference wavelength.

#### 4.5.3. The MTT Reduction Assay

The MTT reduction assay is a colorimetric test that measures the conversion of a water-soluble yellow tetrazolium salt (3-(4,5-dimethylthiazol-2-yl)-2,5-diphenyltetrazolium bromide, or MTT) into insoluble purple formazan crystals by metabolically active cells [96]. After an incubation for 24 or 48 h, the cells’ medium was removed, and warm new medium was added along with a 5 mg/mL MTT solution. Subsequently, the cells were kept at 37 °C for 90 min in the incubator with a 5% CO_2_ flow. In sequence, the medium was removed, and DMSO was added to each well to dissolve the formazans. The plates were shielded from light and placed in a shaker for about 15 min. Ultimately, the absorbance was assessed at 570 nm and 690 nm (reference wavelength) [97], using a Biotek Synergy HT microplate reader (USA). The results were expressed as a percentage relative to the control (set to 100%) after subtracting the values of the reference wavelength.

### 4.6. Mitochondrial Membrane Potential Assay

To evaluate the mitochondrial membrane potential (Δψm), JC-1 was used. JC-1 is a cationic dye that exhibits green fluorescence and accumulates in mitochondria, where it forms red fluorescent J-aggregates in a concentration-dependent manner [98]. In healthy cells with normal Δψm, JC-1 aggregates and emits red fluorescence [98]. However, in apoptotic or damaged cells, decreased Δψm prevents aggregate formation, resulting in green fluorescence [99]. Therefore, the red-to-green fluorescence ratio serves as an indicator of mitochondrial integrity and function [99].

After a 24-h exposure to DOX (0.1 μM, 1 μM, and 10 μM) or SUN (2.5 μM, 5 μM, and 10 μM), Δψm was determined in differentiated SH-SY5Y cells using JC-1. This protocol was previously described by the group in [100]. For fluorescence reading, a Biotek Synergy HT plate reader (USA) was used, and 2 different settings were chosen: 535 nm (excitation)/595 nm (emission) for red aggregates, and 485 nm (excitation)/535 nm (emission) for green aggregates [101].

### 4.7. Western Blot

Given the results for SUN, autophagy was further examined in SUN-exposed cells, focusing on western blot analysis of the autophagy marker microtubule-associated protein 1A/1B-light chain 3 (LC3) forms. LC3 is a key protein in autophagosome formation and a widely used marker for autophagy [71]. Initially, pro-LC3 undergoes proteolytic cleavage by ATG4, generating LC3-I, which encompasses an exposed C-terminal glycine. Through an ubiquitin-like conjugation system involving ATG7 (E1-like), ATG3 (E2-like), and the ATG5-12-16L1 complex (E3-like), LC3-I is lipidated with phosphatidylethanolamine (PE), forming LC3-II. LC3-II is specifically associated with autophagosomes and autolysosomes, facilitating cargo engulfment and degradation [71].

#### 4.7.1. Cell Harvest

Cells were seeded (at the same density mentioned above) in 6-well plates. The cells were exposed to SUN at concentrations of 2.5 μM or 10 μM (concentrations with low and high cytotoxicity, respectively), and both concentrations were incubated with 3-MA or CLQ. Fresh medium was used as the control. Each condition was replicated in 2 wells. The cells were then incubated for 24 h at 37 °C in an incubator with a 5% CO_2_ flow. After the incubation time, the medium was removed and the cells of each condition were scrapped in cold HBSS into a vial, as already described in [46]. To extract the proteins, the vials were centrifugated at 5000 rpm at 4 °C for 5 min and the pellet resuspended in RIPA (150 mM NaCl, 1.0% NP-40 or TritonX-100, 0.5% sodium deoxycholate, 0.1% SDS, 50 mM Tris, pH 8) buffer enriched on that day with 1 mM DL-dithiothreitol, 0.25 mM phenylmethanesulfonylfluoride, and 0.5% (*v*/*v*) protease inhibitor cocktail. The samples were frozen at −80 °C until western blotting analysis.

#### 4.7.2. Protein Quantification

To quantify the levels of total protein in each sample for western blotting, a commercial kit, DC™ Protein Assay (Bio-Rad^®^, Hercules, CA, USA), was used following the recommendations given by the manufacturer.

#### 4.7.3. Electrophoresis and Protein Transference

After the quantification of protein levels in each sample, they were diluted in TRIS buffer (0.5 M; pH = 6.8) to obtain equal protein concentration in all the samples. The samples were reduced using Laemmli buffer [0.5 M Tris-HCl pH 6.8, 4% (*w*/*v*) SDS, 15% (*v*/*v*) glycerol, 0.04% (*w*/*v*) bromophenol blue, and 20% (*v*/*v*) β-mercaptoethanol] and incubated at 100 °C for 5 min [102]. Fifteen μg of protein were loaded and separated on 10% SDS/polyacrylamide gels, at a constant voltage of 175 mV, using a running buffer [100 mL TRIS/Glycine, 10 mL SDS 10%, and 90 mL of water]. Gels were blotted to nitrocellulose membranes for 7 min at 1.3A in transfer buffer [25 mM Tris, 192 mM glycine, and 20% methanol]. Protein loading and transfer were subsequently verified using Ponceau S staining.

#### 4.7.4. Incubation with the Antibodies

To block unspecific sites, membranes were incubated for 2 h at room temperature with agitation in TBS-T buffer [100 mM Tris, pH 8.0, 1.5 M NaCl, and 0.5% Tween 20] containing 5% (*w*/*v*) nonfat dry milk. After that, the membranes were incubated with the primary antibody: anti-LC3 (1:1000) overnight at 4 °C. The next day, the membranes were placed for 20 min at room temperature with agitation, followed by 3 washes of 10 min in TBS-T. The membranes were then incubated with the secondary antibody: anti-mouse (1:10,000). The membranes were, once again, washed 3 times in TBS-T for 10 min, being then incubated with ECL reagents, according to the supplier’s instructions. Images were captured and scanned using the Gel Doc XR system (Bio-Rad^®^, CA, USA) and analyzed with Image Lab software (Bio-Rad^®^, CA, USA, version 6.1). The results of both LC3-I and LC3-II were expressed as the ratio between LC3 and glyceraldehyde-3-phosphate dehydrogenase (GAPDH) levels. For GAPDH, which was used as a loading control, the anti-GAPDH antibody (1:1000) was used on the same membranes following the same washing steps and detection process. The secondary antibody used was anti-rabbit (1:10,000).

### 4.8. Statistical Analysis

Results are expressed as mean ± standard deviation (SD). Outliers were identified and removed following the ROUT test. The data normality was assessed through the Anderson–Darling test, D’Agostino and Pearson test, Shapiro–Wilk test, and Kolmogorov-Smirnov test. All data had a normal distribution in at least one of the previous tests, thus a one-way ANOVA was conducted, followed by the Tukey’s post hoc test when a significant *p* was reached (*p* < 0.05). For the western blot, data were analyzed using a one-way ANOVA followed by Fisher’s LSD test.

The GraphPad Prism 8.3.0 software (San Diego, CA, USA) was used to perform all statistical analyses. Furthermore, all the details of statistical analysis are to be found in the figure’s legends.

Furthermore, the language and grammar of this manuscript were reviewed using Grammarly^®^ (version 14.1237.0, San Francisco, CA, USA) to enhance clarity, coherence, and overall readability.

## 5. Conclusions

In this study, we compared the neurotoxic effects of clinically relevant concentrations of a classical chemotherapeutic agent (DOX) and a targeted therapy (SUN) on SH-SY5Y cells differentiated into dopaminergic neurons. Surprisingly, SUN exhibited a cytotoxic profile like DOX, demonstrating time- and dose-dependent toxicity, along with mitochondrial membrane depolarization. Evaluation of redox homeostasis modulators, such as NAC, DMF, SFN, and CH, revealed varying protective effects depending on the drug and concentration, highlighting potential therapeutic strategies to mitigate cognitive impairments induced by cancer treatment. To better understand SUN-induced toxicity mechanisms, autophagy modulators (3-MA, CLQ, and RAP) were employed. The results suggest that autophagy plays a complex role in SUN-induced toxicity, with the initial phase of the autophagic process providing cellular protection, while later phases are associated with increased toxicity, indicating a disruption in the autophagic pathway. Our study suggests that modulating redox homeostasis, along with autophagy (specifically in the case of SUN), may offer promising therapeutic strategies to mitigate chemotherapy-induced neurotoxicity.

## Data Availability

Data are contained within the article and Supplementary Materials.

## Supplementary Materials

The following supporting information can be downloaded at: https://www.mdpi.com/article/10.3390/molecules30112342/s1.

## Abbreviations

The following abbreviations are used in this manuscript:

3-MA	3-methyladenine
BBB	blood–brain barrier
CH	cheirolin
CLQ	chloroquine
CNS	central nervous system
DA	dopamine
DAT	dopamine transporter
DMF	dimethyl fumarate
DMSO	dimethyl sulfoxide
DOX	doxorubicin
HBSS	Hank’s balanced salt solution
LC3	microtubule-associated protein 1a/1b-light chain 3
MTT	3-(4,5-dimethylthiazol-2-yl)-2,5-diphenyl tetrazolium bromide
NAC	n-acetylcysteine
NR	neutral red
Nrf2	nuclear factor (erythroid-derived 2)-related-factor 2
RA	retinoic acid
RAP	rapamycin
SFN	sulforaphane
SUN	sunitinib
TPA	12-o-tetradecanoylphorbol-13-acetate
VEGF	vascular endothelial growth factor
