# Supplementary material for: Comparative Neurotoxic Effects of Doxorubicin and Sunitinib: An In Vitro Study on Human Dopaminergic Neuronal Cells
Source: Molecules. 2025 May 27;30(11):2342. doi: 10.3390/molecules30112342 (PMC12156406; doi:10.3390/molecules30112342)

1st membrane (image included in the article)

*In all the membranes, the sample are organized in the following order:

CTRL I SUN 2.5 I SUN 10 I SUN 2.5 + CLQ I SUN 2.5 + 3-MA I SUN 10 + CLQ I SUN 2.5 + 3-MA I CLQ I 3-MA

Ponceau

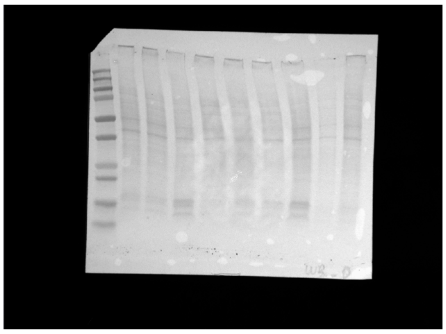

Blot LC3-I (membrane and PW)

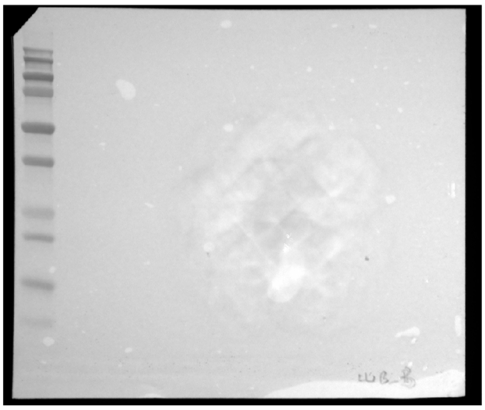

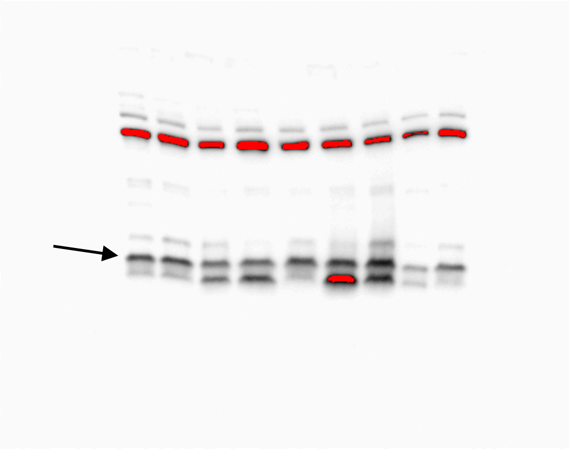

Blot LC3-II (membrane and PW)

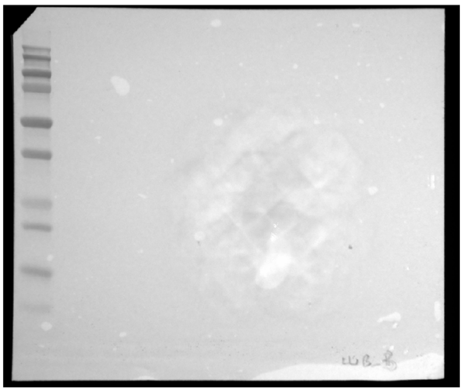

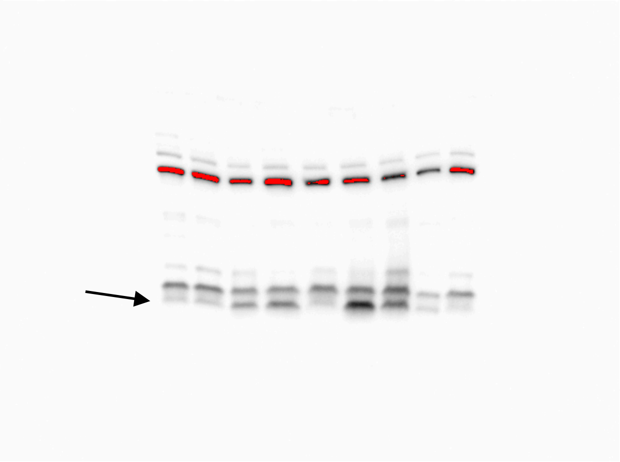

Blot GAPDH (membrane and PW)

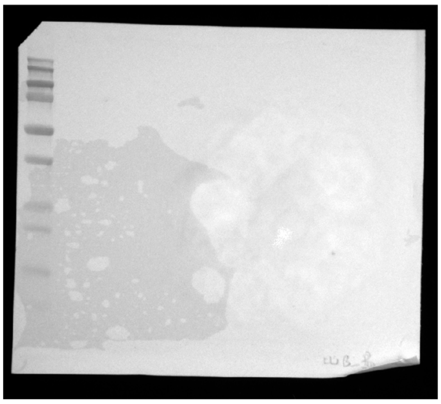

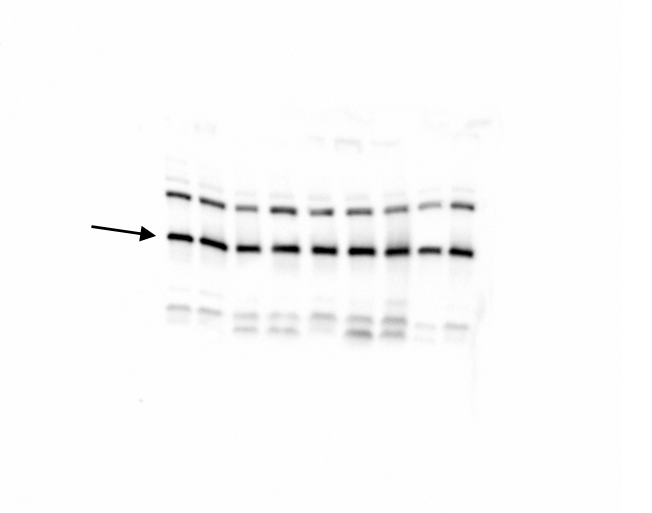

2nd membrane

Ponceau

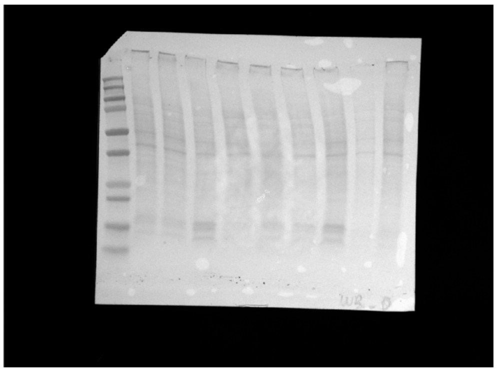

Blot LC3-I (membrane and PW)

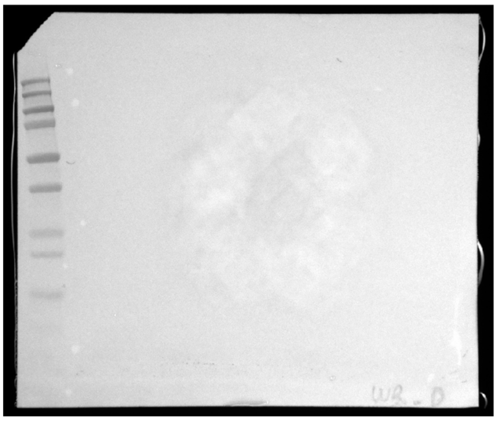

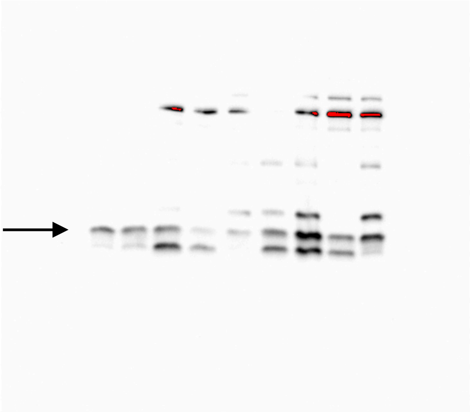

Blot LC3-II (membrane and PW)

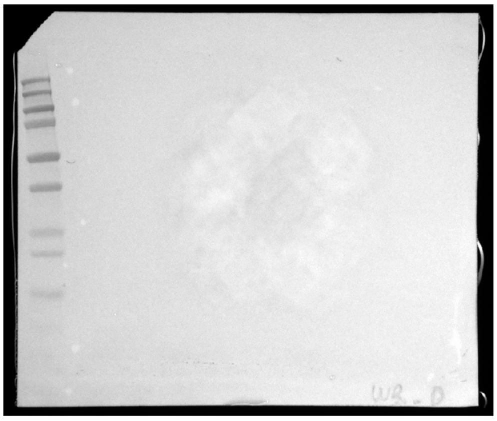

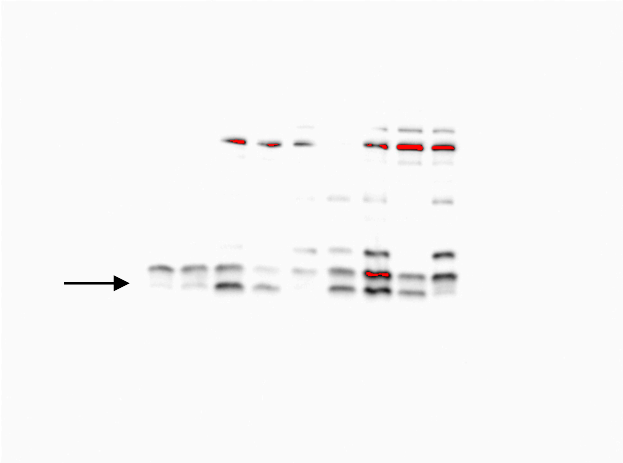

Blot GAPDH (membrane and PW)

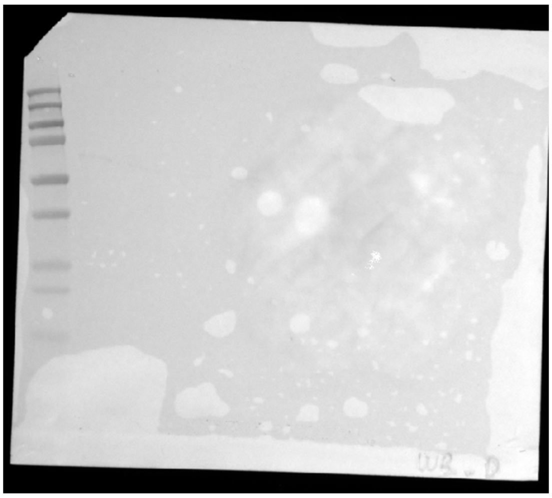

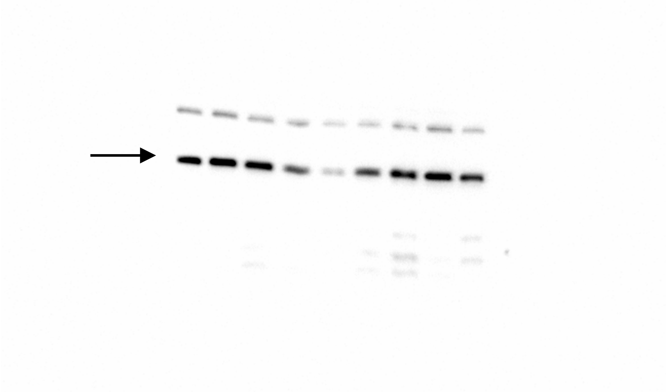

3rd membrane

Ponceau

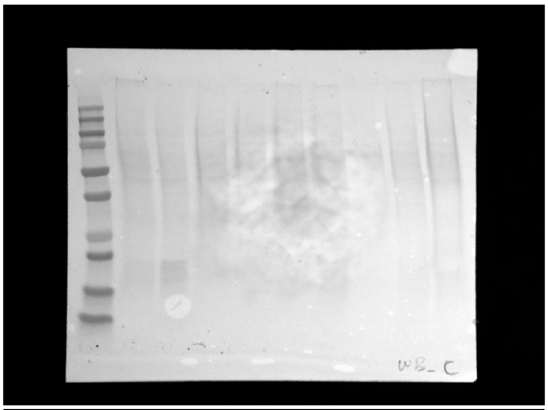

Blot LC3-I (membrane and PW)

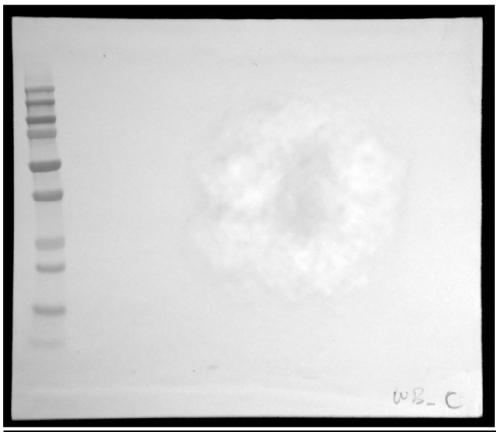

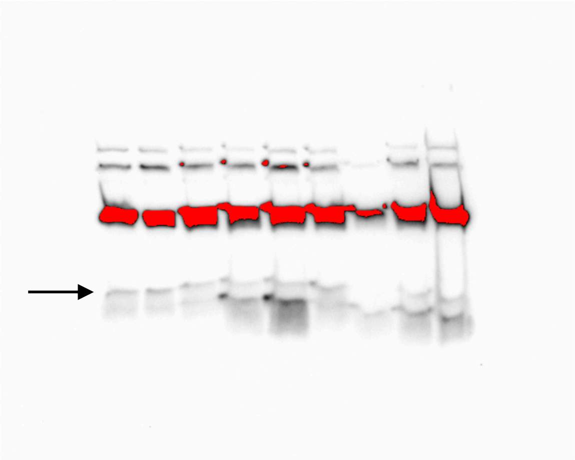

Blot LC3-II (membrane and PW)

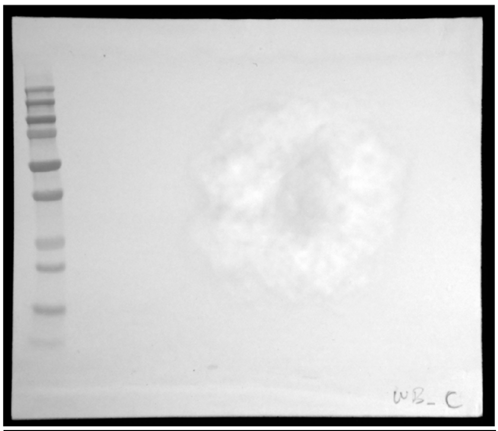

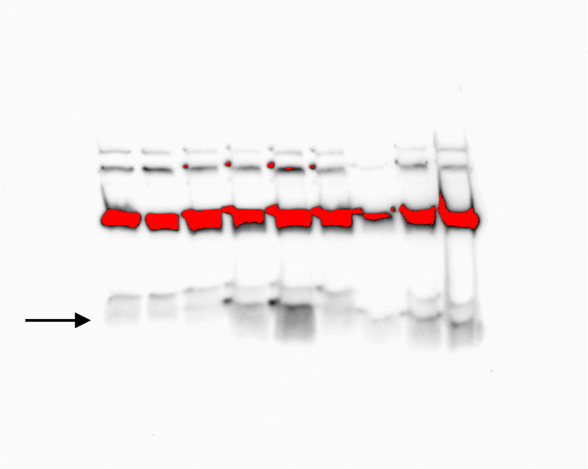

Blot GAPDH (membrane and PW)

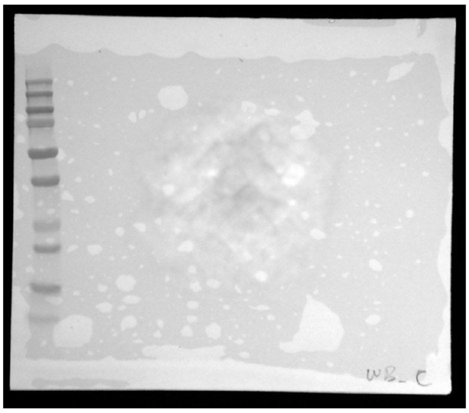

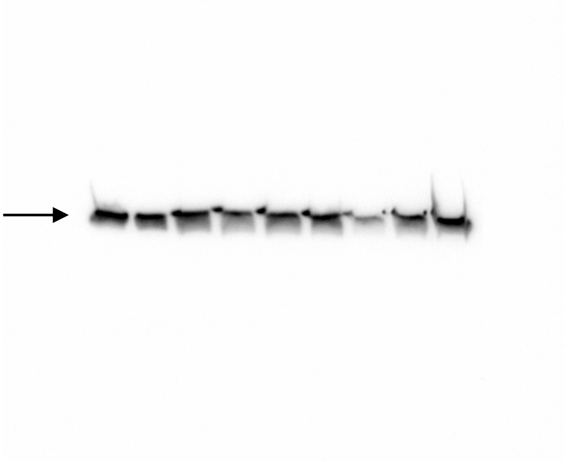

4th membrane

Ponceau

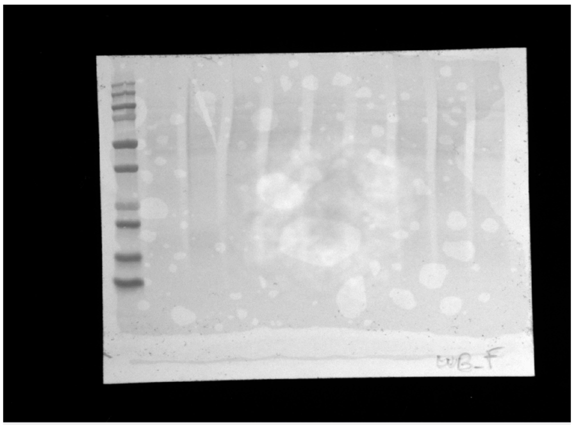

Blot LC3-I (membrane and PW)

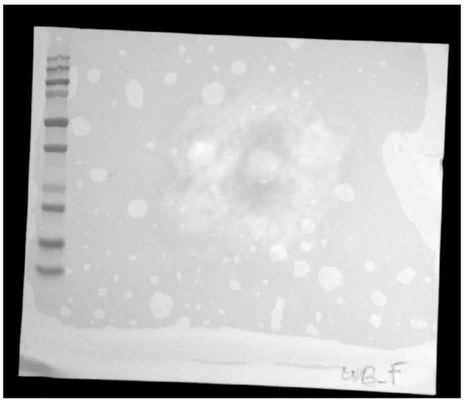

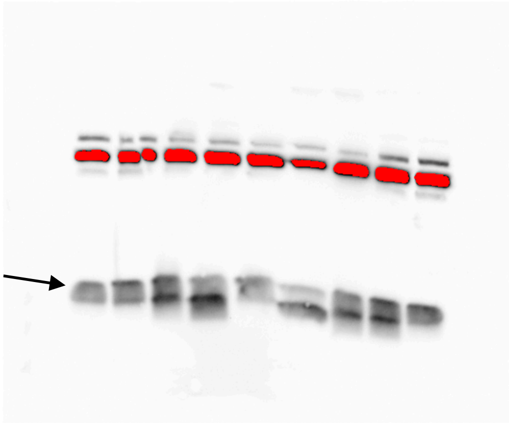

Blot LC3-II (membrane and PW)

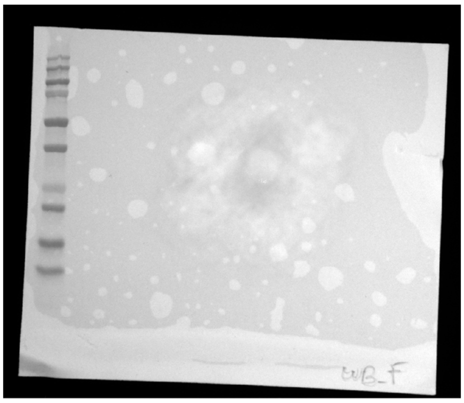

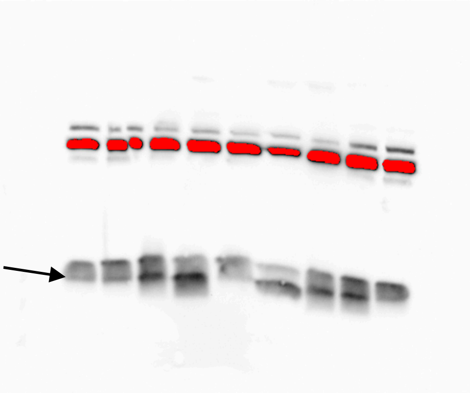

Blot GAPDH (membrane and PW)

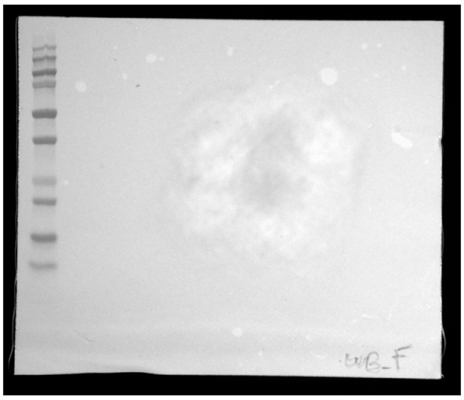

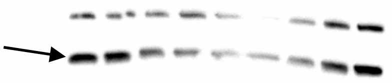

Supplement: Supplementary file 1 [file molecules-30-02342-s001.zip › molecules-3592395-supplementary.pdf]
